# Scaphoid Waist Nonunion in an 8-Year-Old: A Rare Occurrence

**DOI:** 10.1155/2019/4701585

**Published:** 2019-10-14

**Authors:** T. M. Gause, T. E. Moran, J. B. Carr, D. N. Deal

**Affiliations:** Department of Orthopedic Surgery, University of Virginia Health System, Charlottesville, VA, USA

## Abstract

**Case:**

Historically, the most common pattern of pediatric scaphoid injury described is at the distal pole, which has a high rate of success with nonoperative management. Injury patterns have evolved as children are more commonly presenting with adult-type fracture patterns. We present the case of a scaphoid waist fracture in an 8-year-old male that resulted in nonunion and required surgical fixation.

**Conclusion:**

This case highlights the trend of adult pattern scaphoid fractures in the pediatric population and the utility of magnetic resonance imaging in patients who do not have complete carpal bone ossification at the time of initial radiographic evaluation.

## 1. Introduction

Scaphoid fractures are the most prevalent carpal bone fracture in the pediatric population, accounting for 3% of pediatric hand and wrist fractures [1]. The most common age for a pediatric scaphoid fracture is 15 years old, with the earliest documented case reported in a 5-year-old male [2]. Scaphoid fractures are difficult to identify in the pediatric population due to incomplete ossification of the scaphoid at the time of radiographic evaluation. Endochondral ossification of the scaphoid starts at approximately 4 years in females and 5 years in males and is completed roughly by 13 and 15 years, respectively [3].

While nonunion is a well-known complication in the management of adult scaphoid fractures, it is rare in the pediatric population with a reported incidence of 0.8% following closed treatment with immobilization [4]. Several unique characteristics of pediatric scaphoid fractures help prevent nonunion and avascular necrosis (AVN). First, the injury often described is a chondral injury as opposed to a pure bony injury due to the process of endochondral ossification. Second, the traditionally described fracture pattern in skeletally immature patients is a distal pole fracture, which preserves the dominant blood supply. Lastly, these distal pole fractures are usually minimally displaced, allowing for closed management.

Despite the classic description, adult injury patterns are occurring in the pediatric population with increased frequency [5, 6]. This is likely due to increasing sports-related activity and higher energy injuries in children, which subject the scaphoid to stresses similar to adults.

We present the case of an 8-year-old male who sustained a scaphoid waist fracture that was not identified until three years after the initial injury due to lack of ossification on radiographs. The importance of this case is threefold: (1) it is the second youngest case of a pediatric scaphoid waist fracture ever reported, (2) it demonstrates the need to recognize adult pattern scaphoid fractures in children, and (3) it advocates for consideration of advanced imaging in pediatric patients with persistent wrist pain following an injury and in the setting of negative radiographs. Consent was obtained from the patient's parents regarding this case being summarized and submitted for publication.

## 2. Case Report

**Figure 1:**
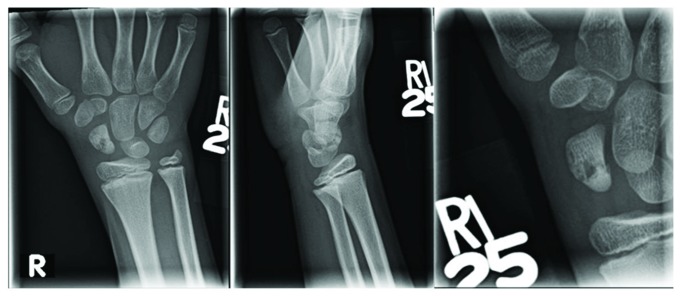
AP and lateral radiographs from November 2016 (approximately 3 years and 1 month from the initial injury) demonstrating a nondisplaced scaphoid waist fracture and a subtle osteosclerotic appearance within the proximal pole. There is also a relative sclerotic appearance of the scaphoid relative to the other carpal bones—a constellation of findings concerning for osteonecrosis [26].

**Figure 2:**
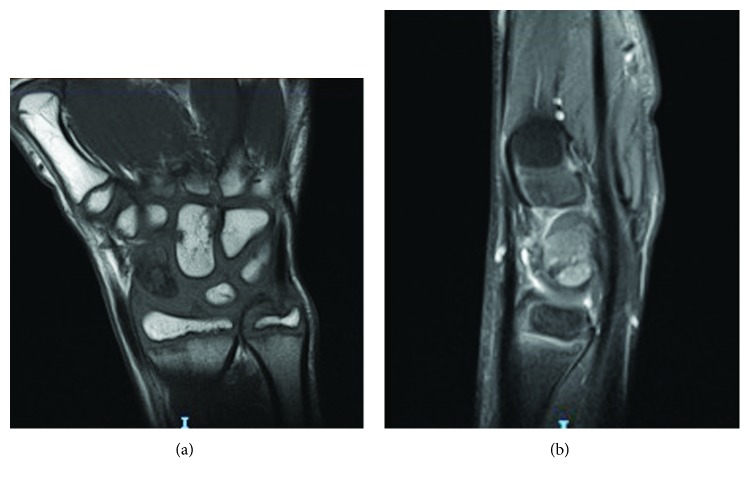
(a) Coronal T1-weighted magnetic resonance (MR) image of the right wrist showing diffuse loss of signal throughout the scaphoid bone that is concerning for osteonecrosis; (b) sagittal fat-suppressed PD-w MR image of the right wrist. The presence of a fracture line is indicative of nonunion.

An 8-year-old male with no significant past medical, surgical, or social history initially presented to an emergency department in October 2013 with a chief complaint of right, radial-sided wrist pain after a fall on his right arm while playing soccer. No fracture was seen on radiographs, and he was placed in a removable splint for comfort and discharged. The patient was scheduled for follow-up but would not present to an orthopaedic clinic until three years post injury, at age 11. At age 11, when the patient was seen at an outside orthopaedic clinic, he reported continued wrist pain from his initial injury three years prior. He described dull pain in his right wrist with intermittent swelling after several minor falls while playing soccer. Radiographs did not reveal a fracture or other pathology, and the patient was again diagnosed with a wrist sprain and given a brace for comfort. He was compliant with the brace, but 8 months later, he again returned to his pediatrician with continued pain. Interval radiographs revealed a right-sided, displaced scaphoid waist fracture (Figure 1). He was referred to our clinic for further management. He was noted to have tenderness located at the anatomic snuffbox but full range of motion. A magnetic resonance imaging (MRI) scan was ordered to further evaluate for AVN and osseous bridging given the chronicity of his symptoms, and he was placed in a thumb spica wrist brace. His MRI demonstrated the scaphoid waist nonunion with concerns for AVN (Figure 2). At this point, the patient and parents elected to undergo surgical fixation of his nonunion. Due to the concern for AVN on MRI, utilization of a vascularized bone graft was considered. However, it was felt that given the young age of the patient, drilling of the bone would be sufficient to stimulate bone growth and avoid the more aggressive procedure.

**Figure 3:**
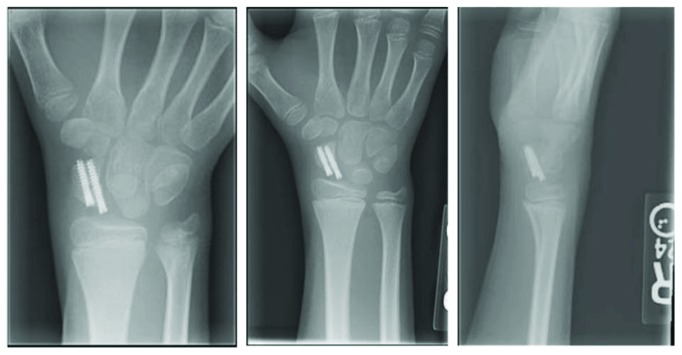
AP and lateral radiographs taken two weeks postsurgical fixation using two Arthrex micro screws.

The patient underwent surgery at 11 years and 3 months of age. Open reduction and internal fixation was performed using a dorsal approach centered over the scapholunate interval, using 14 and 16 mm headless compression screws to achieve fixation. The patient was placed in a thumb spica splint and discharged home the same day. Two weeks later, he presented to clinic with resolution of pain and intact hardware on imaging (Figure 3). He was transitioned to a thumb spica short arm cast. Six weeks postoperatively, he was placed in a removable thumb spica brace for one month. The patient returned to full activity by 4 months postoperatively. One year postoperatively, the patient remained asymptomatic and maintained a full physical activity level without difficulty or pain.

## 3. Discussion

Diagnosing pediatric scaphoid fractures can be difficult, especially before the scaphoid is fully ossified. Previous approaches to managing scaphoid injuries in children were stratified by age, with the assumption that the progressive ossification of the scaphoid predisposes to discrete fractures [7]. Historically, the most common injury was thought to be a distal pole fracture [8–12]. However, recent literature suggests an increasing number of adult injury patterns in children [5, 7]. Stanciu and Dumont reported on a series of 21 patients (mean age of 13 years) who sustained scaphoid fractures. 12 of these patients (57%) sustained scaphoid waist fractures.

The majority of pediatric scaphoid fractures are treated with closed management [4, 10]. Christodoulou and Colton reported successful closed management in 63 of 64 patients aged 8 to 14 years [13]. Surgical treatment is usually reserved for patients who demonstrate nonunion or initial displacement of a waist or proximal pole fracture. The most common method of surgical fixation is screw fixation with or without bone grafting, which has yielded good functional outcomes with regard to range of motion and absence of pain, as well as fusion rates approaching 100% [4, 14–19]. Mintzer et al. reported on 13 scaphoid fractures managed operatively, in which 12 resulted in successful union and 1 requiring revision fixation [17]. Complications following both nonsurgical and surgical treatment are rare with nonunion being the most commonly reported for both treatment modalities [14]. In the setting of failed closed management, surgical treatment is the treatment of choice with excellent outcomes even in the setting of nonunion [19, 20].

The current case report highlights that pediatric patients with wrist pain and negative radiographic imaging should be closely monitored in the acute setting. We recommend that pediatric patients who sustain an injury to the wrist without radiographic evidence of fracture should require close follow-up with consideration of advanced imaging if symptoms do not improve as expected. If wrist pain does not resolve within 6 weeks, it is reasonable to consider advanced imaging modalities such as ultrasound or MRI. MRI is not user dependent and can readily diagnose a scaphoid fracture regardless of the ossification stage. MRI scans can be positive in as little as two days after the injury, and the reported negative predictive value is close to 100%, which may eliminate the need for additional follow-up imaging in the setting of a negative scan [21–25].

Lastly, our study presents an adult type scaphoid waist fracture in an eight-year-old patient, which advocates for greater suspicion for a potentially missed scaphoid fracture in pediatric patients with persistent wrist pain. Furthermore, this case report demonstrates the need for appropriate immobilization in instances of pediatric scaphoid fracture, as it is rare for a scaphoid fracture in a young patient to demonstrate nonunion with proper immobilization. Appropriate immobilization may have been facilitated by the earlier use of advanced imaging for diagnosis of scaphoid fracture in this patient, as scaphoid pathology is difficult to reliably demonstrate radiographically prior to skeletal maturity due to lack of ossification.

## 4. Conclusion

Scaphoid fractures are exceedingly rare within the first decade, particularly in children eight years old and younger. More recent literature shows an increased frequency in identification of adult pattern scaphoid fractures in the pediatric population. Anatomic location of pediatric scaphoid fractures is important since fractures of the scaphoid waist are more susceptible to nonunion and may require prolonged immobilization to achieve union or surgical fixation if there is initial fracture displacement. In patients who are not skeletally mature enough to reliably show scaphoid fractures on radiographs, the use of advanced imaging can aid in identifying scaphoid pathology in cases of unresolved wrist pain and more adequately guide management. In cases of scaphoid nonunion, optimal treatment is controversial, but surgical fixation may be considered and can deliver favorable outcomes. Finally, this case demonstrates that a high index of suspicion for scaphoid fracture must be maintained when treating a pediatric patient with wrist pain and incomplete scaphoid ossification.
